# Effect of Estradiol on Neurotrophin Receptors in Basal Forebrain Cholinergic Neurons: Relevance for Alzheimer’s Disease

**DOI:** 10.3390/ijms17122122

**Published:** 2016-12-17

**Authors:** Andrea Kwakowsky, Michael R. Milne, Henry J. Waldvogel, Richard L. Faull

**Affiliations:** 1 Centre for Brain Research, Department of Anatomy and Medical Imaging, Faculty of Medical and Health Sciences, University of Auckland, Auckland 1142, New Zealand; h.waldvogel@auckland.ac.nz (H.J.W.); rlm.faull@auckland.ac.nz (R.L.F.); 2 School of Biomedical Sciences, Queensland Brain Institute, Clem Jones Centre for Ageing Dementia Research, The University of Queensland, Brisbane 4072, QLD, Australia; milnemichael834@gmail.com

**Keywords:** estradiol 1, neurotrophins 2, neurotrophin receptors 3, basal forebrain cholinergic neurons 4, Alzheimer’s disease

## Abstract

The basal forebrain is home to the largest population of cholinergic neurons in the brain. These neurons are involved in a number of cognitive functions including attention, learning and memory. Basal forebrain cholinergic neurons (BFCNs) are particularly vulnerable in a number of neurological diseases with the most notable being Alzheimer’s disease, with evidence for a link between decreasing cholinergic markers and the degree of cognitive impairment. The neurotrophin growth factor system is present on these BFCNs and has been shown to promote survival and differentiation on these neurons. Clinical and animal model studies have demonstrated the neuroprotective effects of 17β-estradiol (E2) on neurodegeneration in BFCNs. It is believed that E2 interacts with neurotrophin signaling on cholinergic neurons to mediate these beneficial effects. Evidence presented in our recent study confirms that altering the levels of circulating E2 levels via ovariectomy and E2 replacement significantly affects the expression of the neurotrophin receptors on BFCN. However, we also showed that E2 differentially regulates neurotrophin receptor expression on BFCNs with effects depending on neurotrophin receptor type and neuroanatomical location. In this review, we aim to survey the current literature to understand the influence of E2 on the neurotrophin system, and the receptors and signaling pathways it mediates on BFCN. In addition, we summarize the physiological and pathophysiological significance of E2 actions on the neurotrophin system in BFCN, especially focusing on changes related to Alzheimer’s disease.

## 1. Introduction

Estrogens are most commonly associated with their role in control of reproduction, the relative concentrations of the primary estrogen constituents dictating the phases of menstruation and pregnancy [1,2,3]. Estrogens effects are not limited to reproductive regulation [4]. Numerous functions in the central nervous system (CNS) are credited to estrogens, including effects on contextual and spatial learning and memory in cornu ammonis area 1 (CA1) hippocampal and cortical neurons in rodent and nonhuman primate studies [5,6,7,8,9,10]. Estrogens, in particular estradiol (17β estradiol, E2), have also been observed to provide neuroprotection in certain neuronal populations in the CNS, particularly on the cholinergic neurons of the basal forebrain, where E2 has produced ameliorative effects following *N*-methyl-d-aspartate (NMDA) -induced lesions [11,12]. These neurons are often referred to as basal forebrain cholinergic neurons (BFCNs). Several studies have also provided evidence of E2 mediated protective effects on BFCNs by increasing cholinergic neuron function and survival [13,14].

Clinical studies have demonstrated that the incidence of neurodegenerative diseases, including Alzheimer’s disease (AD), are higher in post-menopausal women and this has been attributed to the reduced E2 levels seen in menopause [15,16,17,18]. These findings, along with other experimental results suggest that estrogen therapy may be beneficial in protection against neurodegenerative diseases. Studies have shown that the application of neurotrophin peptides onto BFCNs results in numerous beneficial effects including survival [19] and neuroprotection [20]. One proposed mechanism suggested for neuroprotective effects is that estrogens mediate these effects by influencing the neurotrophin system on neurons of the basal forebrain [13,21,22,23,24]. This hypothesis is backed by evidence from studies indicating that BFCNs express estrogen receptors [23] with further studies suggesting that estrogen receptors are co-localized with neurotrophin receptors on these neurons [24]. Therefore, the anatomical relationship between these two systems provides a platform for the regulation of the neurotrophin system by estrogen in the basal forebrain. Studies investigating the impact of estrogens on neurotrophin peptide and receptor expression have presented various effects. In one such example, long-term estrogen deprivation experiments have shown that significant reductions in tropomyosin-related kinase receptor A were noted in both the medial septum (MS) and nucleus basalis magnocellularis (NBM) of the basal forebrain six months after ovariectomy (OVX) [25]. In in vivo experiments, application of estrogen to ovariectomized rats resulted in an upregulation of BDNF mRNA expression in the cortex and olfactory bulbs, areas linked to BFCNs through afferent innervation and neurotrophic support for these neurons. Furthermore, results of our recent study confirmed that neurotrophin receptor expression in the basal forebrain could be modulated by E2 with effects observed dependent on brain region and neurotrophin receptor [26]. Low levels of estrogen receptor alpha (ERα) were also found to co-localize with neurotrophin receptors in all of the basal forebrain regions examined rising the possibility that E2 acts directly on neurotrophin receptor expression in BFCNs. The use of neuron-specific ERα knockout mice in an attempt to further investigate the role of ERα in this relationship further indicated that ERα is involved in the E2-induced effects on BFCN, as ERα knockout abolished all E2-mediated changes in the neurotrophin receptor expression on BFCN following ovariectomy.

The present review will discuss recent observations into the mechanism of E2 action on the neurotrophin system in BFCN. Furthermore, based on recent experimental findings, we will summarize the physiological and pathophysiological role of E2 in this process.

## 2. Basal Forebrain Cholinergic Neurons

Cholinergic neuron distribution in the central nervous system is widespread including the medial septum, diagonal band of Broca and the striatum [27,28]. These neurons innervate numerous brain areas including the hippocampus, neocortex and amygdala through interactions with a variety of neuron types, including glutamatergic, GABAergic and serotonergic neurons [29,30]. Cholinergic neurons communicate with surrounding neurons via the neurotransmitter acetylcholine (ACh), which is a prominent neurotransmitter in the central and peripheral nervous systems.

The basal forebrain is home to the largest population of the cholinergic neurons in the brain. These neurons provide widespread organized input to regions of the allo- and neo-cortex including the amygdala, hippocampus and cingulate gyrus [31]. Due to their location and afferent and efferent inputs, BFCNs are suggested to be suitably located to monitor and evaluate sensory stimuli and to contribute to cortical activation [32]. This suggests that BFCNs are important in the modulation and maintenance of cortical arousal and attention mechanisms. This has been presented in behavioral studies with results suggesting that BFCNs modulate attention [33,34] and arousal [35,36]. Cholinergic neurons, particularly those in the basal forebrain are vulnerable to a number of neurological diseases with the most notable being AD. Studies have shown that cholinergic markers are reduced in brain tissue of people affected by AD and decreases in cholinergic markers are also linked to the degree of cognitive impairment seen in patients with neurodegeneration [37,38].

### 2.1. Cholinergic Neurons in Specific Basal Forebrain Cholinergic Neuron Subregions

#### 2.1.1. Nucleus Basalis Magnocellularis

The cholinergic neurons which make up the NBM [39] are largest in the basal forebrain [32] and are diffusely distributed throughout the ventral pallidum and substantia innominate [40]. They project to the cortical mantle, the olfactory bulbs and the amygdala [40] which has led to a number of hypotheses on the physiological role of these neurons. In early studies it was believed that NBM cholinergic neurons were involved in learning and memory due to impairments seen when excitatory amino acid lesions were used [41]. However these effects are believed to be attributed to performance decrements, as the use of a selective ChAT inhibitor, which significantly reduced ACh levels in the brain, did not impair ability in the radial arm maze task [42]. Through the use of temporal discrimination tasks [43] and the detection of brief sensory stimuli it has been hypothesized that NBM neurons may be important in aspects of attention [44]. These studies are consistent with the finding that frontal cortical release of ACh is heightened during the anticipation of a predictable reward [45]. Selective destruction of NBM ACh neurons is believed to impair the ability of the neocortex to attend to and process brief, highly salient sensory stimuli [39].

It has also been hypothesized that cholinergic neurons of the NBM neurons are involved in arousal due to the increase in ACh release within the cortex correlating with elevated behavioral arousal [46]. Microinjection of a muscarinic agonist into the NBM was shown to enhance wakefulness, suggesting a NBM specific mechanism [47].

#### 2.1.2. Medial Septum and Diagonal Band of Broca

The medial septum is home to a large cluster of cholinergic neurons. These cholinergic neurons appear smaller than other BFCNs and form a continuous array of multipolar neurons with dendrites that branch out in all directions [48]. These projections innervate the hippocampus, medial prefrontal cortex, retrosplenial cortex and olfactory structures. A neighboring collection of cholinergic neurons to the medial septum is located in the diagonal band of Broca. These cells are slightly larger and irregular compared to those in the medial septum, however their axons project to the same regions as medial septal neurons [32]. Functionally, it has been shown that medial septum cholinergic neurons drive hippocampal theta rhythm, which occurs as a result of phasic inhibition of these cholinergic neurons by GABAergic interneurons. This results in a synchronized rhythmic bursting pattern that drives hippocampal theta activity [49]. Hippocampal theta rhythm is suggested to play a role in short-term memory [50] and therefore it is hypothesized that medial septal neurons in turn play a role in this mechanism.

## 3. Estrogens

Naturally occurring estrogens are C_18_ steroids derived from cholesterol and are similar in synthesis and structure to androgens [51]. All three main estrogens, estriol, estrone and estradiol, share a very similar steroidal four-ring structure with the differences between them located on the D-ring, where they contain either a ketone group, hydroxyl group or two hydroxyl groups. Of these estrogens, estradiol is significantly more potent than estrone and estriol, with a 12-fold higher potency than estrone and an 80-fold higher potency than estriol [52].

Estrogens are synthesized by the aromatase enzyme [53], which catalyzes the aromatization of androgens to estrogens. Androstenedione is aromatized to estrone and testosterone is aromatized to estradiol [51]. Estriol is produced via further hydroxylation of estradiol and estriol [54]. Aromatase is present in a number of tissues, with primary estrogen synthesis occurring in theca and granulosa cells of the ovary and placenta. However, aromatase activity is also present in other tissues including the brain, adipose tissue, liver, fibroblasts and mammary glandular cells [54]. Aromatase expressed in the brain is involved in the control of neuroendocrine events, reproduction and also in the regulation of neural development, synaptic plasticity and cell survival. Aromatase in the brain of vertebrates is produced by neurons and a subpopulation of glia cells [55,56,57,58,59,60]. Aromatase in glia has been found in ependymal cells [61], prefrontal and temporal cerebral cortices [62,63,64] and hippocampus [64,65]. Within the brain, aromatase has been also localized to smooth muscle cells and endothelial cells [65,66].

In men, a small amount of estradiol is produced by the testes and adipose tissue, at levels that are comparable to those seen in postmenopausal women [53]. In women, estrogens have strong control over the reproductive cycle and other processes related to reproduction, like their pivotal role in pregnancy [1,2,67]. Upon synthesis the majority of estrogens reversibly bind to the carrier sex hormone binding globulin which aids in transporting estrogens through the circulation to target tissues [68] while 1%–3% of estrogens circulate in an unbound form until diffusing into target cells to exert cellular effects [69]. Specific estrogens are not produced in equal amounts and the concentrations of these estrogens vary over time, which plays a key role in the modulation of a number of physiological functions. Over the menstrual cycle, estrogen levels fluctuate significantly with estriol and estrone showing the largest variations, at Day 15 these levels peak initiating ovulation and following this their levels remain relatively high, leading to the activation of the luteal phase [3]. E2 on the other hand expresses much lower levels and remains more consistent over the menstruation period [3].

Estrogens have also been implicated in a number of roles in the body not related to reproduction. They act as natural vasoprotective agents and in smooth muscle cells and blood vessel endothelial cells. Estrogens have been shown to promote vasodilation and reduce smooth muscle tone [70] via effects on nitric oxide release and calcium channel modulation. These non-reproductive effects of estrogens might explain why the incidence of cardiovascular disease is lower in females. Estrogens effects on bone growth and strength have also been demonstrated. Both osteoclasts and osteoblasts express estrogen receptors [71,72]. Estrogens directly inhibit bone degradation caused by osteoclasts and a deficiency in estrogens is believed to indirectly result in the stimulation of osteoclast differentiation [73]. Estrogen deficiency has been shown to accelerate bone loss and increase susceptibility to fractures, while estrogen therapy has been observed to reduce bone weakening [74].

## 4. Estrogen Receptor Signaling Pathways

Physiological and cellular responses to estrogens and all other steroid hormones are linked to receptors that initiate a vast array of biological pathways upon estrogen binding. In the case of estrogen receptors, these responses have been divided into two categories based on response type: classical and non-classical responses, also known as genomic and non-genomic responses, respectively. Genomic responses are characterized by activation of the classical estrogen receptors resulting in a signaling cascade that ends with gene transcription and takes from hours to days for an effect to occur. Non-genomic responses on the other hand are illustrated as rapid signaling events which can occur within seconds of receptor activation. Rapid responses are often G protein-coupled receptor (GPCR) mediated and promote responses such as ion flow and kinase activation. Contrary to their names, activation of the non-genomic pathways also results in gene transcription via second messenger pathways. However, only the classical response pathway results in the activation of the estrogen response element (ERE) transcription regulator.

### 4.1. Estrogen Receptors

**Figure 1:**
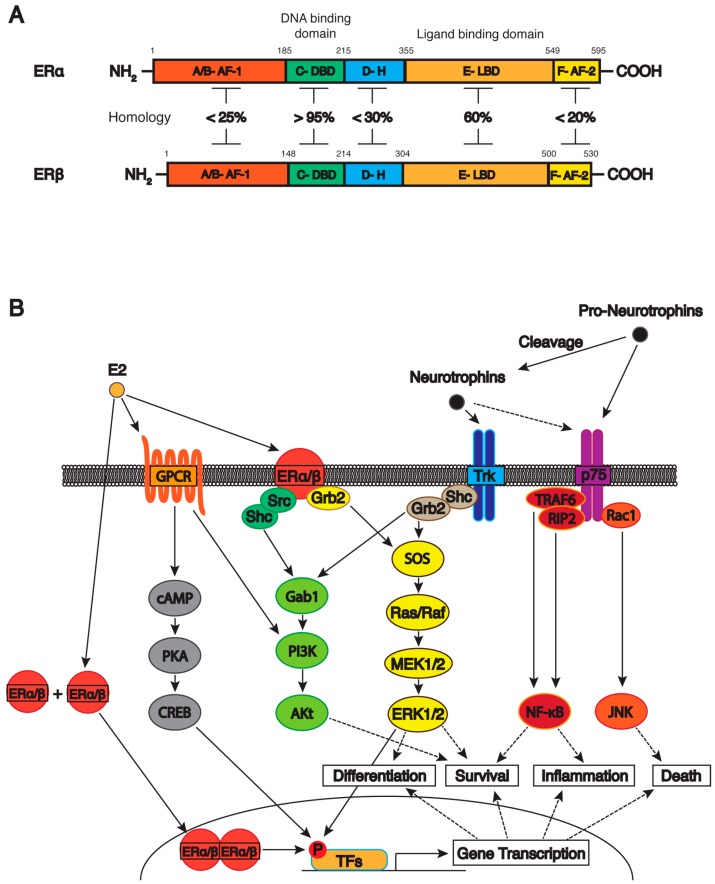
(**A**) Estrogen receptor structure and function. Homology between ERα and ERβ: amino acid identity (%) in the N-terminal activation function 1 region (AF-1), DNA-binding domain (DBD), hinge region (H), ligand-binding domain (LBD), and C-terminal function 2 domain (AF-2); (**B**) interaction of neurotrophin receptor and estrogen signaling pathways. Schematic outlining of the neurotrophin receptors and their associated peptides and a simplified diagram describing the main signaling pathways activated by the Trk and p75 receptors. Activation and subsequent phosphorylation of the Trk receptor results in the activation of the MEK/ERK signaling pathway, that leads to phosphorylation of transcription factors (TFs) like CREB and promotes cell differentiation and survival; and the PI3K/Akt pathway that promotes cell survival. The p75NTR signals modulate the NF-κB and JNK pathways, which promotes inflammation and cell survival or apoptosis respectively. The classical and non-classical estrogen pathway interacts with neurotrophin system and regulates neuronal survival and ameliorative effects on the BFCN. Dashed arrows indicate various targets.

Estrogen receptors are a class of ligand activated transcription factors and belong to the steroid hormone superfamily of nuclear receptors, which also includes receptors for progesterone, corticosteroids and testosterone. There are two classical estrogen receptors, the first of which is estrogen receptor α (ERα) which was discovered in 1958 [75]. Initially, ERα was believed to be crucial for development and survival. It was later discovered that mutations leading to ERα knockout did not significantly impact survival [76] and further studies using ERα knockout mice found that specific estrogen binding could still be observed [77]. These discoveries suggested the existence of a second estrogen receptor. In 1996, the cDNA for a second estrogen receptor was isolated and subsequently named estrogen receptor β (ERβ) [78]. The discovery of a second estrogen receptor raised questions about the specific structural and functional differences of these receptors. ERα is encoded by the *ESR1* gene on human chromosome 6 and ERβ is encoded by *ESR2* on chromosome 14 [79], therefore making each receptor a product of a completely different gene [80]. Yet, both receptors share a high level of homology, with >95% amino acid homology in the DNA binding domain and 60% amino acid homology in the ligand binding domain [81] (Figure 1A).

Both receptors share a similar overall structure consisting of a highly conserved DNA-binding domain that contains two zinc fingers important for in DNA binding and receptor dimerization as well as a partially conserved ligand binding domain, involved in ligand binding, receptor dimerization and nuclear localization [82]. Receptors also contain a variable N-terminal, a hinge domain and C-terminal that are involved in activation of target genes, contribution of flexibility to DNA/ligand binding domains and the transactivation capacity of the receptor, respectively [82] (Figure 1A).

Estrogen receptor expression varies depending on tissue type and both receptors exhibit distinct pattern of expression. Studies in the rat have shown that ERα has higher levels of expression in the uterus, adipose tissue, testis, bone, pituitary, ovary theca cells, kidney, mammary gland, epididymis and adrenal gland while ERβ expression is strongest in the prostate, ovary granulosa cells, colon, lung immune system, heart, and bladder [78,83,84].

Estrogen receptors also show strong expression in the CNS, ERα can be found in the ventromedial hypothalamic nucleus and subfornical organ while ERβ is located in the olfactory bulb, hypothalamus, cerebellum and tegmental area. Areas which express both estrogen receptors include the amygdala, preoptic area, periaqueductal grey and locus ceruleus [85]. Although the general distribution is similar, data cannot be extrapolated from one species to another. For example, the paraventricular nucleus of rats is reported to express only ERβ while in mice it contains ERβ and some ERα positive cells and in humans both are present but with higher concentrations of ERα [86]. In the mouse central nervous system, the level of cytoplasmic ERα or ERβ in the dorsal hippocampal region varied with the estrous cycle [87]. Furthermore, in synapses of the rat hippocampal CA1 region, both ERα and ERβ decreased with age but, in contrast to ERα, the expression of ERβ increased in response to E2 in older animals [88]. In many cell types, ERα and ERβ form either homodimers or heterodimers. They are found in nuclear, cytoplasmic and membrane sites throughout the brain. ERs are expressed on neurons as well as glia, which are known to play a major role in providing nutrient supply for neurons and involved in regulation of systemic metabolism and energy balance [89]. Astroglia, microglia and oligodendrocytes are all target cells for estrogen receptors [90]. In addition, estrogen receptors are expressed and modulate gene expression and produce long-term effects on neurovascular endothelial and smooth muscle cells [91,92].

In terms of receptor function, ERα and ERβ have been shown to have strong and very distinctive functions. Specifically, ERα has a more profound effect on the development and function of the mammary gland and uterus and on the maintenance of metabolic and skeletal homeostasis. It has been shown that deletion of the *ERα* gene in female and male mice results in infertility, gonadal deformities and decreased bone density, suggesting estrogen receptors are involved in the development of the gonads and the maintenance of fertility [77]. ERβ, however, has more pronounced effects on the central nervous system and on conditions of cellular hyper proliferation. Both receptors have important roles in the development and function of the ovaries and in the protection of the cardiovascular system.

### 4.2. Classical Estrogen Signaling Pathway

The classical estrogen pathway involves the direct modulation of gene transcription by estrogens via estrogen receptors. Due to the steroid structure of estrogens, they can pass freely through the plasma membrane of target cells into the cytosol and nucleus where they can interact with estrogen receptors. Upon binding of estrogens to ERα or ERβ a conformational change occurs which promotes the homodimerization of estrogen receptors and removal of regulatory receptor associated proteins [93]. In the absence of estrogen, estrogen receptors associate with co-repressor molecules that act to inhibit transcriptional activity of the receptor. The binding of estrogen promotes a conformational change in the ER leading to dissociation of the co-repressors and attachment of co-activators, which act to increase its transcriptional ability at ERE sites on specific genes [94,95]. In the nucleus estrogen receptors function as ligand dependent transcription factors by binding to ERE located in the promoter region of target genes. This promotes the recruitment of specific co-regulators which can increase or decrease the rate of transcription of target genes [96]. Transcription leads to alterations in protein levels in the cell thereby producing changes in cell functionality. Activation of estrogen receptors also leads to indirect activation of gene transcription rather than binding directly to target genes estrogen receptors can interact with other transcription factor complexes including Fos/Jun or SP-1 [97] to influence transcription of genes lacking EREs.

### 4.3. Non-Classical Estrogen Signaling Pathway

The first evidence of non-traditional estrogen receptor mediated pathway was presented in a study in which acute 17β-estradiol administration could produce rapid increase in cAMP concentrations in the uterus [98]. This pathway has been defined as the “non-classical” estrogen receptor pathway. ERα and ERβ proteins lack known functional motifs that could facilitate non-genomic mechanism of activation via estrogen. However, several studies have suggested that ERα and ERβ mediate rapid estrogen stimulated activation via second messengers [11,99]. These rapidly-initiated biological responses are mediated by binding of estrogens to plasma membrane bound or cytosolic receptors. Estrogens can mediate their non-classical effects via caveolae-localized estrogen receptors, estrogen receptor-X, G protein-coupled receptor 1 and non-steroid diphenylacrylamide receptor [100,101,102,103,104]. A candidate receptor believed to be involved in mediating this rapid process is the plasma membrane bound G protein-coupled receptor 30 (GPR30) [105]. GPR30 expression was found in ER-positive human breast tumor cell lines and in endocrine tissues [105,106]. Further studies have supported the hypothesis of a estrogen-mediated membrane bound G protein-coupled receptor; in one such study demonstrating that 17β-estradiol is successful in activating ERK-1 and ERK-2 and adenylyl cyclase in cells lines expressing ERα and ERβ (MCF-7) and also in breast cancer cell lines (SKBR3) which do not express either receptor but do express GPR30 [107]. The effect of GPR30 on ERK activation was investigated using a breast cancer cell line that does not express GPR30 (MDA-MB-231); ERK in these cell lines was not activated by subsequent 17β-estradiol application. However, forced overexpression of GPR30 in this cell line resulted in the activation of adenylyl cyclase in the presence of estrogen [108]. There have been additional studies that have also demonstrated that rapid estrogen action upon cells does not require ERs. This has been shown in experiments involving ER antagonists which stimulate adenylyl cyclase activity in human MCF-7 breast cancer cells [109]. Pharmacokinetic studies have provided further evidence that the ER independent signaling pathway activation is via a GPCR. This has been demonstrated by an increase in [^35^S] GTP binding to the plasma membranes of SKBR3 and HEK-293 cells expressing GPR30 in the presence of estrogen [110]. Further evidence suggested that GPR30 is attached to a stimulatory G protein (Gs) as estrogen treatment resulted in an increase in immunoprecipitation of activated G-proteins to antibodies specific to the GPR30 α_s_ subunit [110,111]. GPR30 is expressed by neurons, glia and can also be found on the neurovasculature [89,112,113].

The cellular effects mediated by the non-classical pathway can be achieved through direct action on ion channels or through the activation of intracellular second messenger cascades [114,115]. It has been shown that E2 binding at estrogen receptors can activate both PI3K and the MAPK pathways and PKC to a lesser extent. Activation of these second messenger pathways can carry out a number of cellular effects including phosphorylation of cAMP response element binding (CREB) which in turn initiates indirect gene transcription. Activation of these pathways has also been shown to be important in a number of physiological and neuroprotective functions including activation of ERK1/2. This can lead to increased β-amyloid precursor protein secretion, which decreases β-amyloid protein generation. Furthermore, estrogen mediated PI3K activation protects against light induced photoreceptor degeneration [116].

In addition, these non-classical estrogen receptor mechanisms have been shown to occur in BFCNs. In a study by Szegő and colleagues in 2006, it was shown that acute 17β-estradiol treatment in OVX mice resulted in a rapid increase in CREB phosphorylation in a number of basal forebrain regions. This effect was demonstrated to be mediated by ERα and involve the MAPK and PKA secondary messenger pathways [117]. These results indicate the complicated nature of estrogen signaling in the CNS and the vast array of resultant downstream effects that can occur as a result of this (Figure 1B).

### 4.4. Neuroprotective Effects of Estrogens in the Central Nervous System

Studies of estrogens effects on cholinergic neurons in the basal forebrain were among the first to suggest that estrogens can also provide non-reproductive actions. Studies involving ovariectomy and estrogen replacement therapy demonstrated the involvement of estrogens in regulating ChAT expression, with studies showing that estrogen treatment increased ChAT expression in projection areas of the basal forebrain 10 days after estrogen injection [118]. Cholinergic dendrites have been known to decrease in length in the absence of estrogen, as observed in OVX mice. This reduction in dendritic length seen in OVX mice was successfully reversed in OVX mice when estrogens were reintroduced through an E2 releasing capsule [119].

In the hippocampus, an area highly involved in contextual and spatial learning and memory, estrogens have been observed to positively affect memory in animal models and in humans [118]. These beneficial effects of estrogens are believed to be linked to increases in dendritic spine density seen on CA1 pyramidal neurons following estrogen treatment in ovariectomized mice [120]. Other proposed effects related to estrogen stimulation include regulation of the serotonin system, which is involved in the regulation of autonomic nervous system reactivity to mood and cognitive function [4].

A number of other neuroprotective roles of estrogen in the CNS have been hypothesized. Several studies have reported estrogen to be neuroprotective in brain ischemia models. In one such study investigators reported a regional protective effect against ischemia induced cell death [121]. Estrogen implants of E2 have also been shown to reduce lesion size in male rats which were subjected to middle cerebral artery occlusion [122]. In this study, investigators discovered that following right middle cerebral artery occlusion OVX rats which had been implanted with an E2 releasing capsule at the time of OVX had significantly reduced overall infarct volume compared to vehicle treated controls and that this protective effect was specific to the cortex with no protection seen in the striatum. Acute estradiol administration at the time of ischemia failed to protect against ischemic injury, suggesting that long-term presence of estrogens is needed to provide neuroprotection [121]. These results have been attributed to actions on ERα as studies on ERα and ERβ knockout mice have found that neuroprotective effects of estradiol are abolished in ERα knockout mice but not ERβ knockout mice [123].

Neuroprotective effects have also been noted in the basal forebrain, where estradiol has produced ameliorative effects in BFCNs following NMDA induced lesions [11]. E2 administration provided a restorative effect to BFCN fibers projecting to the cortex. Interestingly, it has also been shown that aromatase activity in brain areas damaged by kainic acid or physical trauma is increased which could suggest that a physiological mechanism is in place increasing local estrogen concentrations to provide a neuroprotective effect at sites of neuronal damage [124]. Further studies have suggested that estrogens may be beneficial in protection against neurodegenerative diseases, most notably AD. E2 significantly ameliorates the cholinergic neurodegeneration in experimental rodent models of AD [11,125,126,127,128]. Brain and plasma levels of E2 have been reported to be lower in women with AD as compared to an age-matched control group. This is noted especially in women with menopause. However, clinical studies into the effect of estrogens on the incidence of AD are conflicting [129,130].

AD is characterized by the accumulation of Aβ plaques and tau neurofibrillary tangles, and early loss of cholinergic neurons. Other factors may also contribute to the pathophysiology of AD, such as mitochondrial dysfunction, oxidative stress, neurotransmitter failure and inflammation. One proposed mechanism for estrogens protective effects against AD is that E2 can regulate amyloid-β (Aβ) accumulation through activation of the estrogen receptor [131]. In terms of Aβ accumulation, E2 is involved in both Aβ production and clearance. E2 promotes the non-amyloidogenic cleavage of APP by increasing the expression of α-secretase and it promotes Aβ clearance by increasing the internalization and phagocytosis of Aβ by microglia [131,132,133,134,135]. Recent studies have suggested that beneficial effects of estrogen on AD are directly linked to its ability to reduce not only Aβ but also tau aggregates. Estrogens can alter the post-translational modification of tau, through phosphorylation of glycogen synthase kinase-3β (GSK-3β) and protein kinase A (PKA) [136]. It is possible that abnormal accumulation of Aβ and tau may originate from a common cause and that tau may be an important effector of Aβ-mediated neurotoxicity or they may also exert synergic effects in the disease pathology. Estrogens ability to stimulate ChAT expression in the BFCNs in animal models [137,138] has been suggested to delay the decline BFCN function in estrogen replacement therapy in aging and AD. This may delay the degeneration of BFCNs and slow cognitive decline into AD. However, several other positive effects of estrogens can be attributed to its powerful antioxidant, anti-apoptotic, and neurotrophic activities.

## 5. Neurotrophins

Neurotrophins are a family of growth factor proteins that regulate the development, survival and function of a large population of compatible neurons in the central nervous system. The functions of the neurotrophin receptors vary markedly from the regulation of development of the nervous system to the regulation of survival and regeneration in damaged neurons. Downstream effects of neurotrophin receptors are complex, with some neurotrophin signals activating, mitogenic or survival responses while others activate apoptotic signals, with the type of response dependant on neuron type, location and neurotrophin peptide/receptor combination. This combination of mitogenic and apoptotic signaling results in a system capable of finely regulating the activity of neurons in both a positive and negative manner depending on the present factors.

In mammals there are four peptides which make up the signaling molecules of the neurotrophin family; nerve growth factor (NGF), brain-derived neurotrophic factor (BDNF), neurotrophin 3 (NT-3) and neurotrophin 4 (NT-4). Neurotrophins are initially synthesized as precursor proteins called pro-neurotrophins which are cleaved intracellularly by the endopeptidase furin or pro-convertases to form their mature forms [139]. These neurotrophins act via two types of neurotrophin receptor; the p75 receptor and the family of tyrosine kinase receptors. There are three tyrosine kinase receptors; tropomyosin-related kinase receptor A (TrkA), tropomyosin-related kinase receptor B (TrkB) and tropomyosin-related kinase receptor C (Trk C). TrkA binds NGF, TrkB binds BDNF and NT-4, and TrkC binds NT-3 [140]. The p75 receptor (p75) can bind to each neurotrophin with a greater affinity for the pro-neurotrophins and can also act as a co-receptor for Trk receptors [141]. In the brain neurotrophin receptors exhibit a highly exclusive expression with the majority of receptors found in the basal forebrain, with studies suggesting TrkA, TrkB and p75 expressed in the basal forebrain and striatum co-localize strongly with cholinergic neurons [26,142].

Neurotrophin receptor expression has been extensively studied in neuronal cells but their expression and function in glial cells is more controversial in the brain. However, BDNF and its receptor TrkB are present in Bergmann glia and BDNF stimulation is linked to the activation of the phosphatidyl-inositol 3 kinase/protein kinase C/mitogen-activated protein kinase/Activator Protein-1 signaling pathway [143]. Astrocytes also express TrkB and both release and recycle neurotrophins [144]. Besides neurons diverse CNS cell types express the neurotrophins NT-3, NT-4 and BDNF, such as astroglia, oligodendrocytes, and microglia [145]. p75 has also mainly been studied in neurons but it is expressed in a variety of glial populations, especially during development and after injury [146].

### 5.1. Neurotrophin Receptors

Neurotrophin peptide responsive neurons interact with neurotrophins via transmembrane glycoproteins, of which two classes are known to exist. These are a tyrosine kinase class of receptors which are members of the Trk family and the single binding protein related to CD40 receptors known as the low affinity neurotrophin receptor or p75 neurotrophin receptor [147]. Each of the Trk receptors interacts with one or more of the neurotrophin peptides and the localization of these receptors correlate with specific neurotrophin responsive tissues. This correlation led to the discovery of the first Trk neurotrophin receptor, TrkA. Links between NGF stimulation of tyrosine kinase activity and the distribution of a Trk protooncogene was similar to NGF responsive neurons led to the discovery that receptor p140trk was the NGF receptor [148]. Thus, TrkA has been found in NGF responsive neuronal tissues such as sympathetic and sensory neurons [149]. The presence on neurons promotes a number of pro-growth effects, expression of TrkA in mouse 3T3 cells lines results in enhanced anchorage independent growth, survival and cell proliferation in the presence of NGF, with weaker effects observed in the presence of NT-3/4 [150].

Following the discovery of TrkA, cDNA library screens led to the isolation of structural homologs of TrkA which were subsequently named TrkB [151] and TrkC [152]. TrkB is a receptor for BDNF and NT-4/5 and is expressed on BDNF responsive tissues throughout the CNS mainly on sensory ganglia and motor neurons. Studies have shown that when TrkB and TrkC are expressed on 3T3 fibroblasts the appropriate neurotrophins yield functional responses that are similar to those obtained with TrkA. TrkB is activated equally by BDNF and NT4/5 and to a lesser extent by NT3 [153]. TrkC is expressed on cells responsive to NT-3; these include spinal sensory neurons and adrenergic neurons of the locus coeruleus [147].

The p75 neurotrophin receptor (p75NTR) was isolated around the same time as TrkA. The existence of p75NTR first became apparent due to studies which observed NGF responsive receptors on various neuronal and tumor cell lines which had heterogeneous binding affinity for NGF, this lead to the hypothesis that there were high and low affinity NGF receptors. Purification of these receptors yielded a 130–140 kDa and a 75–80 kDa protein, the 130–140 kDa protein was TrkA and the 75–80 kDa protein was named p75NTR or low affinity neurotrophin receptor. Studies into the pharmacodynamics of p75NTR showed that it had similar affinities for all neurotrophin peptides [154]. Pro-neurotrophins are more selective ligands for the p75NTR than their mature neurotrophin forms and are more effective at inducing p75NTR -dependent apoptosis [155].

p75NTR is the neurotrophin receptor which is the most poorly understood due to the more complicated nature of this receptor and its mechanisms of action. This mainly due to complex interactions with Trk receptors in which, it can act directly with Trk receptor and have altered signaling capacity depending on the state of other Trk receptor [141]. The most well documented role of p75NTR is its ability to mediate neuronal apoptosis [156], which it has been shown to mediate in; sympathetic neurons, motor neurons, sensory neurons, oligodendrocytes and Schwann cells [157]. Studies suggest p75NTR only mediates apoptosis when Trk receptors are inactive or suboptimally activated leading to the conclusion that Trk receptor activation silences apoptotic signaling [158]. This provides neurotrophins with the ability to stimulate survival in Trk receptor positive neurons and promote apoptosis in neurons lacking Trk receptor.

### 5.2. Neurotrophin Peptides

The primary influential peptides that act upon neurotrophin receptors to alter cellular functions including apoptosis and differentiation are the neurotrophin peptides. Of these, NGF was the first discovered and is the best characterized of the known neurotrophin peptides. NGF was discovered as a diffusible substance with the ability of stimulating growth of sympathetic and sensory spinoganglia cells but not of motor neurons via production by target organs and tissues, providing trophic support to developing neurons [159]. Since its discovery additional NGF responsive cells include lymphocytes [160], mast cells [161] and eosinophils [162]. Following the discovery of NGF it was suggested that the majority of neurons in the CNS are responsive to NGF-like factors, this led to the isolation of the second neurotrophin peptide, brain-derived neurotrophic factor (BDNF). Studies provided evidence that BDNF promoted the survival of sensory neurons, but not sympathetic neurons [163]. Furthermore, some types of ganglia in the CNS that were unresponsive to NGF specifically, but still relied on neurotrophic factors, were found to be influenced by BDNF. Structural studies of BDNF reported that it was structurally similar to NGF (~50% similarity), suggesting the two peptides were related [164]. This provided the groundwork for the discovery of two additional neurotrophins; neurotrophin-3 (NT-3) and neurotrophin-4/5 (NT-4/5) via protein structure studies focusing on the conserved sequences of NGF and BDNF [149,165].

### 5.3. Role of the Neurotrophin System in Neuroprotection

The first discovered Trk receptor activated signaling protein shown to mediate survival was the GTP binding protein Ras. Inhibition of Ras activity decreased survival of most, but not all populations of sympathetic neurons which are sensitive to neurotrophins [166]. Conversely, deletion of a Ras regulatory inhibitor resulted in an increase in Ras activity allowing peripheral neurons to survive in culture in the absence of neurotrophins [167]. Ras activity is postulated to direct neurotrophin activation signals into multiple signaling pathways. The two major pathways believed to be involved are the PI3K/Akt and MEK/MAPK pathways, which are the key effectors of neurotrophin induced survival [168,169] (Figure 1B).

PI3K was first identified as a regulator of neurotrophin mediated survival responses in NGF dependent PC12 cells [170]. It was shown that Ras directly interacted with PI3K and that inhibition of Ras suppressed NGF mediated PI3K activation. Further studies have indicated that PI3K activates downstream Akt to mediate neurotrophic effects, for example adenovirus incorporation of dominant-negative Akt resulted in a 40% reduction in NGF induced survival of sympathetic neurons [171]. One of the mechanisms mediated by Akt to induce survival is by stimulating Ca^2+^ influx through L-type calcium channels [172]. Akt has been shown to only play a role in survival and not in neurite outgrowth or differentiation.

The other pathway that is activated by Trk receptors is the Ras-MEK-MAPK pathway; this pathway has roles in synaptic plasticity, long-term potentiation and survival which is believed to be due to downstream increases in cAMP-response element binding transcription factor activity [173]. This pathways involvement in Trk signaling is more complex however as results have shown that while NGF induces a strong and sustained activation of MAPK in PC12 cells and sympathetic neurons, inhibition of MEK has minimal effects on NGF dependent survival [174]. Suggesting that MEK/MAPK can promote cell survival, but are not necessary for neurotrophin mediated survival under most circumstances. It has also been hypothesized that the major role of this pathway may be to protect neurons from cell death as a result of injury or toxicity [157].

The signaling pathway mediated by p75NTRs to initiate apoptotic mechanisms is less understood and involves a number of pathways including JNK, NF-κB and ceramide [175]. A number of studies have implicated the involvement of Jun amino-terminal kinase (JNK)-p53-Bax, which has been shown to be activated via p75NTR activation and following NGF withdrawal [158]. p53 appears to be the key death sensor in this pathway as levels of this protein in cells correlates strongly with apoptosis. Another factor found to be activated by p75NTR is the transcription factor which has been shown to be activated in Schwann cells [176], oligodendrocytes [177] and sensory neurons [178]. Unlike the JNK-p53 pathway, activation of the NF-κB pathway by p75 is not blocked by activation of TrkA [179]. Studies have indicated that this pathway appears to represent a p75 mediated pro-survival pathway that collaborates with Trk receptors. One such study showed that treatment of sympathetic neurons with NGF led to NF-κB activation that was important for NGF mediated survival [180]. It has been suggested that this synergism between Trk receptor and p75 may be a result of a dual activation of Akt’s survival promoting activity in cells where p75 can associate with TRAF6 which leads to NF-κB activation [181].

Due to the varied nature of effects p75NTR activation can produce (Figure 1B), it has been concluded that the signaling capacity and role of p75NTR is highly dependent on the activation status of nearby Trk receptors. With the most simplistic association being that Trk receptor signaling silences the apoptotic mechanism mediated by p75NTR, without affecting other p75-mediated pathways, such as NF-κB activation. It is suggested that Trk receptor may act through Ras-PI3K/Akt to interfere with p75NTR induced apoptosis by suppressing the JNK-p53-Bax pathway upstream of JNK [157]. Conversely, p75NTR also appears to have modulatory effects over Trk receptor activation. Studies have shown that p75NTR may act as a modulator of Trk receptor-induced neuronal growth, which may be useful for regulating the specificity and density of axonal growth and target innervation [182]. It is believed that p75NTR activation causes selective downregulation of the TrkA-dependent Raf-MEK-MAPK pathway, which is a major growth pathway for sympathetic neurons [157].

NGF-mediated signaling through TrkA is reduced in AD patients. While the precursor form of NGF (pro-NGF) that selectively binds to p75NTR to promote cell death is increased in the disease [183,184]. Furthermore, p75NTR s are expressed in Trk receptor negative, degenerating neurons also in AD brains only [185]. Neurotrophins can also regulate the expression and cleavage of the amyloid precursor protein [186] and Aβ is capable of binding to p75NTR s [187]. However, due to complexity of p75NTR signaling, it is not clear if Aβ toxicity is mediated by p75NTR in the AD brain and how p75NTR contribute to the etiology of the disease [188]. Importantly, the BDNF Val66Met polymorphism has been demonstrated regulating synaptic excitation and neuronal integrity, being the only genetic factor that is able to moderate the effects of Aβ on memory decline and hippocampal atrophy, both in sporadic and autosomal dominant AD [189,190].

### 5.4. Neurotrophins in the Basal Forebrain

The basal forebrain is considered to have the highest expression and be greatly influenced by the neurotrophin system. In terms of NGF distribution in the CNS in-depth analyses of its distribution focusing mainly on cholinergic neurons of the basal forebrain showed that brain areas either containing BFCN cell bodies or BFCN innervation have the largest expression of NGF [191]. Other areas containing cholinergic neurons, including the striatum, and areas not expressing cholinergic neurons showed significantly lower levels of NGF [191]. Similar to NGF expression, studies have also shown that BDNF has a similar pattern of distribution, with studies demonstrating high expression in the major areas of the basal forebrain and also in regions of the CNS are known to be innervated by BFCNs including the hippocampus, amygdala and neocortex [192].

Studies investigating the role of neurotrophins in the basal forebrain have discovered a number of effects that this system is responsible for. Neurotrophin peptides including NGF and BDNF have been shown to promote survival and differentiation of basal forebrain neurons in in vitro studies. A lesion in the fimbria-fornix, which connects the septum to the hippocampus, prevents the retrograde transportation of NGF from the hippocampus to the basal forebrain, which results cholinergic cell loss and atrophy [19,193], while intracerebrovascular (i.c.v) administration of NGF into the basal forebrain following lesion prevents this cholinergic neurodegeneration [194,195]. NGF appears to be the most potent of the neurotrophin peptides in its effects on BFCNs, being shown to be particularly effective at influencing the number and size of these neurons [196]. Neurotrophins effects on BFCNs have been observed to promote effects other than survival and differentiation, with a number of studies reporting that NGF increases not only the survival rate of cholinergic neurons but also enhances ChAT enzyme activity [193,197]. This suggests that the neurotrophin receptors are not only involved in BFCN development and survival but are also involved in BFCN functionality.

The neurotrophin peptide BDNF has also been implicated in the maintenance of BFCNs. In one such study investigators demonstrated the ability of an i.c.v injection of BDNF to significantly reduce degenerative changes to cholinergic cells in the septal area of the basal forebrain following fimbria axotomy [198]. This result is similar to that seen in other lesion studies using NGF, in which NGF provided a neuroprotective effect when introduced following lesion [194]. When the efficacy of NGF and BDNF were compared, BDNF was found to produce a weaker neuroprotective effect than NGF in the basal forebrain [198].

Neurotrophin receptors in the basal forebrain have been studied extensively to uncover their role in this area, with multiple effects being discovered. One such study that investigated the role of TrkA and NGF in BFCNs observed anatomical and behavioral changes in transgenic mice lacking TrkA in the BFCN [199]. Results showed that there was a profound deficit of stable cholinergic innervation to the cortex and hippocampus, with a significant proportion of these innervating axons failing to connect and successfully innervate target areas observed. No observable change was seen in survival rate of these neurons. A reduction in ERK activation and ChAT expression was also seen in the basal forebrain. These neurological changes were accompanied by mild cognitive deficits in learning and attention tests. Furthermore, these mild cognitive defects seen are characteristic of early stage AD, providing evidence for the involvement of neurotrophin system dysfunction in this pathology [199].

Studies have suggested that the p75NTR is also critically involved in the basal forebrain, with evidence that its effects are more pronounced than that of the Trk receptors. Receptor expression studies have stated that more than 90% of cholinergic neurons in the basal forebrain are p75 receptor positive and that p75 receptor expression is reserved exclusively to cholinergic neurons in the basal forebrain [26,200,201].

Other studies investigating the role of the p75NTR in the basal forebrain have investigated the influence of p75NTR actions on neuronal features including neuronal size, neurotransmitter synthesis and target innervation [202]. In p75 knockout mice, the absence of p75 caused an increase in neuronal size and ChAT activity. This supports the hypothesis that p75 negatively regulates the trophic system in BFCNs. Furthermore, a lack of p75NTR resulted in an increased innervation of the hippocampus by these neurons, further reinforcing the negative role of p75NTR in the basal forebrain, through its involvement in axonal pruning. p75NTR knockout did not however affect the gross structure of the basal forebrain cholinergic system, which is similar to results seen in NGF and TrkA knockout mice [203]. Other studies have investigated the apoptotic nature of p75NTR, one such study focused on the effects of neurotrophins and pro-neurotrophins in BFCNs [204]. Results showed that pro-NGF and pro-BDNF were effective in inducing apoptosis of cultured basal forebrain neurons, suggesting an effect by p75NTR. Furthermore, it was shown that during injury pro-neurotrophins could be produced by astrocytes, leading to caspase mediated cell death in basal forebrain regions. This effect was linked to the p75NTR as the use of a p75NTR blocker prevented cell death.

## 6. Relationship between Neurotrophins, Estrogens and AD in the Basal Forebrain

Clinical studies have demonstrated that the incidence of neurodegenerative diseases, including AD, are higher in post-menopausal women and this has been attributed to the reduced E2 levels seen in menopause [15,16,17,18]. These findings, along with other experimental results suggest that estrogen therapy may be beneficial in protection against neurodegenerative diseases. One proposed mechanism for estrogens protective effects against neurodegenerative diseases is that E2 influences the neurotrophin system on neurons of the basal forebrain to provide neuroprotective effects, with studies suggesting that estrogen receptors are co-localized with neurotrophin receptors on these neurons [13,21,22,23,24] (Figure 1B).

Cholinergic neurons, particularly those in the basal forebrain are vulnerable in AD. Due to the wide spread neurodegeneration seen in the AD brain and the complex underlying pathomechanism, the exact mechanisms that lead to the degeneration of BFCNs is not yet understood. The time course of cholinergic deficit and whether the neuropathological changes are primary or secondary to cortical pathology are unknown. Furthermore, some studies show significant neuronal atrophy in the NBM rather than cell death [205], others found high variability between patients [206,207], while some reports suggest up to 90% cell loss [208]. Recent high-resolution MRI imaging studies have shown that basal forebrain cholinergic system volume in some cases began declining in early adulthood, the atrophy aggravated in advanced age and exacerbated in AD from the earliest stages of cognitive impairment [209]. Another recent study showed that atrophy of the NBM is associated with structural changes of their innervated regions in prodromal AD and women with amnestic mild cognitive impairment showed a stronger association between volume of the NBM and thickness of the temporal lobe when compared with healthy age-matched women [210]. Studies have shown that cholinergic markers are reduced in brain tissue of people affected by AD and decreases in cholinergic markers are also linked to the degree of cognitive impairment seen in patients [37,38]. In addition, significant shrinkage of cholinergic neurons, axonal degeneration and synaptic loss have been reported in AD patients [205,211]. In in vivo mouse models of AD, we found a 37% Aβ_1-42_-induced loss of cholinergic neurons in the NBM and a 30% loss of cholinergic fibers from the somatosensory cortex. A single injection of E2 30 min following Aβ_1-42_ administration was able to restore loss of cholinergic cortical projections but did not prevent the loss of cholinergic neurons in the NBM [128].

Examination of cholinergic neuron populations in the MS, NBM and striatum of ovariectomized mice and ovariectomized mice treated with E2 indicated that the average neuron number in each region is unaffected by two weeks OVX [26]. Thus, this study confirmed that changes in neurotrophin receptor expression following OVX did not occur as a result of changing cholinergic neuron number. These results are in agreement with other studies indicating that OVX did not result in a reduction of cholinergic neuron population in the MS and NBM of rats over a maximum time period of one month [212]. Further studies indicated, however, that long-term loss of ovarian function of six months but not three months resulted in a significant decrease in cholinergic population size in the MS and NBM [213]. Furthermore, BFCN number and size also decreases with age starting early in adult life in both animals and humans [214,215].

There have been various investigations over the past few decades into the effects of estrogens on neurotrophin systems, in particular neurotrophin receptors located on BFCNs. The main goal of these studies has been to determine if estrogens apparent beneficial effects in AD occur as a result of changes produced to the neurotrophin system on cholinergic neurons in the basal forebrain which are particularly vulnerable to AD [216]. After many years of study however a definitive answer has still not been reached, with studies providing a wide range of theories on the role of estrogens on neurotrophin receptors in the basal forebrain [13,25,217,218]. A short 7–10 days treatment with physiological doses of estrogens resulted in a significant increase of ChAT and trkA mRNA in the rat NBM after ovariectomy that decreased both parameters [218,219,220]. In the mouse NBM a reduced number of TrkA, TrkB and p75NTR -expressing choline acetyltransferase-positive cells were observed in ovariectomized mice. In animals with E2 replacement, the TrkA, TrkB, and p75NTR expression was comparable with the expression levels found in the ovary intact group. Long-term estrogen deprivation studies have shown a significant reduction in TrkA receptor mRNA in both the MS and NBM 6 months after OVX [25]. Further studies have confirmed a link between E2 and neurotrophin receptors, with results in other studies observing a 38% decrease in TrkA receptor mRNA expression in the NBM and band of Broca of the rat basal forebrain 10 days following OVX which was reversed with short-term E2 replacement seven days after surgery [219].

The literature is controversial regarding the presence of ERα and ERβ in the BFCNs in animals [138,220,221]. It is hypothesized that the neuroprotective effects of estrogens are mediated via ERα, as ERα is the predominant E2 receptor expressed in the basal forebrain of rodents [222]. Studies in ERα and ERβ knockout mice have found that neuroprotective effects of estradiol are mediated by ERα [11,123,128]. Our recent study aimed to provide a greater understanding of the effects of E2 on neurotrophin receptors on cholinergic neurons in different basal forebrain regions and demonstrated the complex nature of the relationship between E2 and the neurotrophin receptors. We found that E2 influences neurotrophin receptor expression on BFCNs with effects depending on neurotrophin receptor type and neuroanatomical location and the effects require possibly both indirect and direct mechanisms trough ERα in BFCNs [26]. We found that the co-localization of ERα in BFCNs with neurotrophin receptors, TrkA, TrkB and p75NTR, in mice is between 6% and 19% only but ERα knockout, using neuron-specific estrogen receptor-α knockout mice, abolished all E2-mediated changes in the neurotrophin receptor expression on BFCNs. Several studies provide strong evidence that ERα is highly involved in the protective mechanisms of E2 in the basal forebrain and in other brain regions [11,13,123], which could also mediate the preservation of neurotrophin receptors on cholinergic neurons in the basal forebrain. The neuron specific knock out of ERα in a NMDA lesion mouse model prevented the neuroprotective effects of E2 in the NBM of mice [11]. ERα deletion also abolished the protective effects of E2 after ischemic brain injury [123]. The role of ERβ in E2-mediated neuroprotection has also been investigated, but results suggest that only ERα is required to protect neurons [13]. Thus, ERα is an important mediator of E2s neuroprotective effects on the BFCNs. New evidence continues to suggest that modulation of neurotrophin receptor expression by E2 may account for these protective effects and E2 therapy might be beneficial in neurodegenerative conditions, like AD.

Studies have demonstrated minor changes in ERα and ERβ immunorectivity in the human NBM with aging but a significant increase of both receptors in AD [223]. This study suggests that ER availability is not a limiting factor for the effect of estrogen replacement therapy. However, it also indicates that estrogens’ effect on cognition might be mediated by other basal forebrain nuclei or brain areas [223].

Alterations in NGF level, TrkA, TrkB and p75NTR within the NBM in early AD when there is still no cholinergic cell death suggest that the neurotrophin signaling is required for the survival of these neurons [224,225,226]. The degeneration of NBM neurons is associated with decreased neurotrophin responsiveness and E2 therapy could possibly upregulate neurotrophin receptor expression, improve cholinergic and cognitive function.

## 7. Conclusions: Therapeutic Potential of Estrogens for AD Treatment and Prevention

Estrogens have been stated to have a wide therapeutic potential with benefits being seen in naturally occurring estrogens as well as synthetic and plant derived phytoestrogens. Endocrine therapies, first used more than 100 years ago, are one of the most effective treatments for estrogen receptor positive breast cancer [227]. Hormonal supplementation in peri- and postmenopausal women is a promising preventive or treatment option for neurodegenerative conditions such as AD [228]. However, due to side effects, including heightened risk of breast cancer, coronary heart disease and stroke, associated with hormone replacement therapy (HRT), it remains a source of great health concern [229,230]. To avoid these, it is critical to set the appropriate time when HRT is initiated and terminated, the treatment regime, dosage, formulation and combination of HRT [231,232]. It is accepted that hormone replacement therapy should be initiated during menopausal transition or soon after the beginning of menopause [130]. This “window of opportunity” or “critical period” will also allow neurons that are still relatively healthy to benefit from estrogen exposure [233]. On the contrary, women who have lived 10–20 years in a hypo-estrogenic state have accumulated neuronal damage below the critical threshold that cannot be repaired by estrogen therapy. Recent data suggest that the timing of HRT might lead to different response on the neurotrophin system and E2 therapy should be initiated during the critical period while there are no changes in neurotrophin receptor expression levels, the neurons are still healthy and able to benefit from exogenous hormonal exposure [26,233]. Moreover, the time and differences in chronological and reproductive aging has to be taken into consideration as well. The symptoms of neurological disorders, like seizures and migraine, change in frequency when E2 levels fluctuate [234]. During menopause, estradiol levels change dramatically, therefore many alterations occur in the female brain as serum E2 levels fall. One of the challenges to understand the role of E2 and its effects is that the targets of estrogens are diverse and neurotrophin receptors are just a few of these. However, as the population ages and the prevalence of age-related neurological conditions like AD increases and the development of interventions that optimize cognitive aging are of critical importance. Based on the evidence reviewed above, estrogens or estrogen-like compounds, like the activators of nongenotropic estrogen-like signaling (ANGELs) used for hormone replacement therapy that may potentially eliminate the unwanted HRT-related side effects, might be one of these interventions [235]. Estren is a gender-neutral synthetic steroid with beneficial effects on bone health, cognition and promising “AD” therapeutic potential [128,235,236].

The expression of Trk receptors has been studied in the NBM of AD patients and, interestingly, the proportion of neurons expressing TrkA, TrkB and TrkC shows a differential reduction [237]. While our studies demonstrated a significant increase in TrkA and TrkB receptor levels in astrocytes and plaques in the CA1 region of the human hippocampus [238], the fact that neurotrophin receptors show differential regulation by E2 depending on brain region and the type of neurotrophin receptor just adds to the complexity of the possible outcomes [26]. Therapeutic interventions demonstrated very limited efficacy for AD. A possibility that a combinational therapeutic approach might show more potent results has been proposed for several drugs. One option is that addition to HRT neurotrophin therapy early in the disease process could potentially prevent the cholinergic neuronal loss in the basal forebrain. This can also have further positive effects, like enhancement of Ach synthesis and fiber growth [239]. HRT can regulate neurotrophin receptor expression but neurotrophin therapy could ensure the appropriate level of their ligands in the basal forebrain. NGF does not pass the blood-brain barrier and local delivery is needed, that limits its therapeutic potential. Intracranial infusion of NGF can improve cognition but patients develop side effects [240]. However, the problem was overcome by the use of encapsulated NGF biodelivery to the basal forebrain [241,242]. This approach is well tolerated; improves cognition, nicotine binding, and electroencephalogram activity; results in less brain atrophy; and slows down the rate of atrophy [243].

In summary, more research is needed to define these parameters that can influence the successful outcome of HRT on the neurotrophin and cholinergic system in the brain. Further studies are needed to investigate the molecular pathways that involved mediating the effects of E2 and neurotrophins on the cholinergic system and their interactions in order to design the best possible effective therapies for AD.
